# Evaluation of Post-Operative Morbidity and Palatal Wound Healing after Implant Uncovering Surgical Procedure Performed with Apically Positioned Flap (APF) and Leukocyte and Platelet-Rich-Fibrin (L-PRF): An Original Technique

**DOI:** 10.3390/medicina60010096

**Published:** 2024-01-04

**Authors:** Giuseppe Balice, Luca Bettocchi, Imena Rexhepi, Matteo Serroni, Luigi Romano, Bruna Sinjari, Paolo De Ninis, Giovanna Murmura, Michele Paolantonio, Beatrice Femminella

**Affiliations:** 1 Department of Innovative Technologies in Medicine and Dentistry, University “G. d’Annunzio” Chieti-Pescara, 66100 Chieti, Italy; giuseppe.balice2@gmail.com (G.B.); luca.bettocchi@gmail.com (L.B.); matteo.serroni@unich.it (M.S.); luigi.romano@unich.it (L.R.); b.sinjari@unich.it (B.S.); michele.paolantonio@unich.it (M.P.); beatrice.femminella@yahoo.it (B.F.); 2 “Luisa D’Annunzio” Institute for High Culture, 65123 Pescara, Italy; paolodeninis@gmail.com

**Keywords:** palatal wound healing, apically positioned flap, leukocyte and platelet-rich-fibrin peri-implant surgery, second stage implant surgery

## Abstract

*Background and Objectives*: Dental implants are recognized as an effective treatment in the management of edentulous patients; controversies surround the connection between the sufficiency of keratinized gingiva (KG) and peri-implant health. Maintaining an ample amount of peri-implant KG is crucial for minimizing gingival inflammation, highlighting the need for regular consideration of soft-tissue augmentation. Among the diverse periodontal plastic surgical procedures, the apically positioned flap (APF) is notable for its ability to enhance the width of keratinized tissue while minimizing patient morbidity. The aim of this study was to evaluate the effects of L-PRF on palatal wound healing and patient discomfort after surgery. *Materials and Methods*: Twenty patients with two adjacent submerged fixtures in the maxilla and buccal keratinized gingiva widths < 2 mm were treated with APF and L-PRF. Clinical evaluations were performed at 1, 2, 3, and 4 weeks post-surgery, focusing on parameters such as complete wound epithelialization (CWE), postoperative discomfort (D), changes in feeding habits (CFH), alteration of sensitivity (AS) around the wound area, and the consumption of analgesics. *Results*: Our data revealed CWE in 5 patients by the end of the second week, with the remaining 15 achieving CWE by the end of the third week. For D and CHF, a statistically significant improvement was recorded for all cases between the first and second weeks, as well as AS, although less substantial, by the third week. No significant changes were noted for AS over the initial two weeks. *Conclusions*: These findings suggest that L-PRF may enhance wound healing and decrease patient discomfort following APF for fixture uncovering.

## 1. Introduction

The use of dental implants has proven to be a successful approach to treating edentulous patients. The achievement of osseointegrated implants primarily hinges on factors such as the preservation of both hard and soft tissues and adherence to appropriate surgical and prosthetic procedures. The maintenance of healthy peri-implant soft tissue is crucial for establishing an effective seal between the oral environment and the implant, including its associated superstructure, thereby significantly contributing to the long-term success of dental implants. It is noteworthy that the protective barriers surrounding an osseointegrated implant may face challenges, as the parallel arrangement of gingival connective tissue fibers can compromise the strength of the peri-implant mucosal seal. Although current evidence is still inconsistent on the importance of having a sufficient amount of keratinized tissue (KT) to prevent peri-implant inflammation and to increase implant survival [1,2], several studies have shown that an adequate band of masticatory mucosa is associated with a lower plaque index, less bleeding on probing around implants, and less discomfort during home oral hygiene procedures [3,4]. Moreover, the amount of keratinized mucosa will also influence prosthetic emergence profile, biological width, and aesthetics [5].

Keratinized mucosa preservation, or its increase, may be obtained at the same implant placement phase, at the submerged fixture uncovering phase, or during the restorative procedure. In implant dentistry, the clinician can choose between two different implant methods: the “one-stage” method, in which the transmucosal part of these implants is integrated with the fixture, and the “two-stage” method, in which the fixture is submerged under the mucosa and the transmucosal part is connected to the implant in a second surgical procedure. The latter implant method avoids bacterial contamination and also prevents undesirable loading of the implants during the osseointegration period [6]. Therefore, it is advisable to contemplate soft-tissue augmentation to establish a keratinized mucous zone that maximally encompasses the implant prosthesis. The management of soft tissue in regions undergoing implant rehabilitation can be conducted either prior to the surgical stage, following the surgical phase and preceding the prosthetic phase, or after the conclusion of the prosthetic phase. Among surgical uncovering techniques for biphasic implants, the apically positioned flap (APF), consisting of the buccal displacement of the keratinized mucosa located palatally to the fixture, has proved to be effective in creating keratinized mucosa buccal to the implant or increasing the amount [7]. However, healing of the area palatal to the healing abutment, from which the tissue (keratinized epithelium and part of the chorion) has been buccally displaced, will occur by secondary intention, increasing the risk of post-surgical bleeding, discomfort, and morbidity for the patient [8]. In order to improve post-operative recovery, many authors suggested the use of hemostatic medication for wound covering, such as absorbable synthetic collagen, ferric subsulfate solution, absorbable gelatin sponge, and oxidized regenerated cellulose [9,10]. Numerous studies have stated the efficacy of leucocyte platelet-rich fibrin (L-PRF) in promoting the healing of surgical wounds and reducing post-operative patient discomfort [11,12].

L-PRF is a second-generation platelet concentrate with a tridimensional fibrin network enclosing platelets and leucocytes capable of releasing growth factors and cytokines [i.e., transforming growth factor-ß (TGF-ß), platelet-derived growth factor (PDGF), vascular endothelial growth factor, epidermal growth factor, insulin growth factor-1, interleukin-1ß, inteleukin-4, interleukin-6, tumor necrosis factor-α], promoting neo-angiogenesis, proliferation of fibroblasts, and migration of epithelial cells [13]. These properties would determine faster wound closure, better hemostasis, and rapid remodeling of scar tissue [14]. The purpose of this study is to evaluate healing time and post-operative discomfort after implant uncovering using APF associated with L-PRF bandages at the palatal surface of the wound.

## 2. Materials and Methods

### 2.1. Study Population

Approximately 20 patients (13 males), aged 35–65 years, with a total of 40 maxillary implants, were recruited from 54 candidates. They were treated in the Unit of Periodontology and Dental Hygiene of the ‘G. D’Annunzio’ University of Chieti-Pescara between April 2021 and May 2022. For each patient, only one surgical site was considered.

The inclusion criteria were: (1) not having systemic diseases that could interfere with the surgical treatment; (2) not being pregnant or lactating; (3) being a non-smoker; (4) having a full-mouth plaque score (FMPS) and a full-mouth bleeding score (FMBS) < 20% at surgery; (5) having been subjected to the application of two adjacent two-stage fixtures, positioned in the maxillary arch and covered by soft tissue that, in relation to the mid-line of the edentulous area, had less than 2 mm of keratinized mucosa; (6) not having hypersensitivity to Ketoprofen.

The volunteers signed a consent form approved by the ethical committee of G. D’Annunzio University after having received comprehensive information. This study was performed according to the principles of the Helsinki Declaration, revised in 2013.

### 2.2. L-PRF Preparation

**Figure 1:**
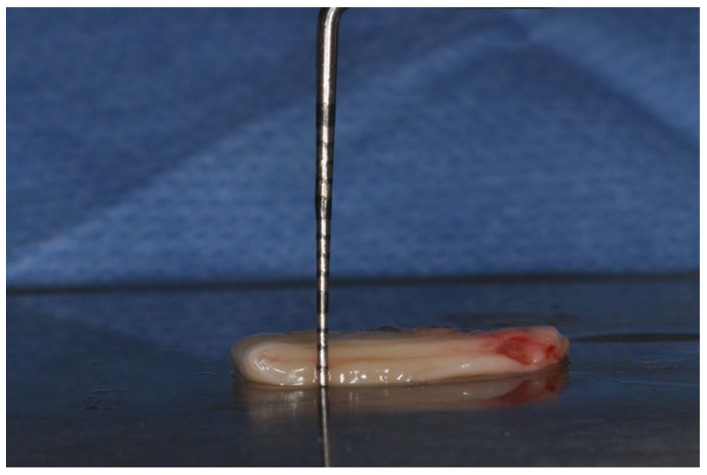
Two membranes were superimposed to create a double layer of L-PRF.

L-PRF was prepared according to the protocol developed by Choukroun et al. [15] Immediately before surgery, a venous blood sample was taken from the antecubital vein and collected in two 10 mL sterile tubes without anticoagulant. Subsequently, it was quickly centrifuged (IntraSpin™, Intra-Lock System Europa SpA, Salerno, Italy) at 3000 rpm for 10 min. The fibrin clot was collected and squeezed in an L-PRF Box (Xpression™ Fabrication Kit, Intra-Lock System Europa SpA, Salerno, Italy) in order to obtain two L-PRF membranes; for each patient, the membranes were superimposed to create a double layer of about 2 mm thickness (Figure 1).

### 2.3. Surgical Procedure

**Figure 2:**
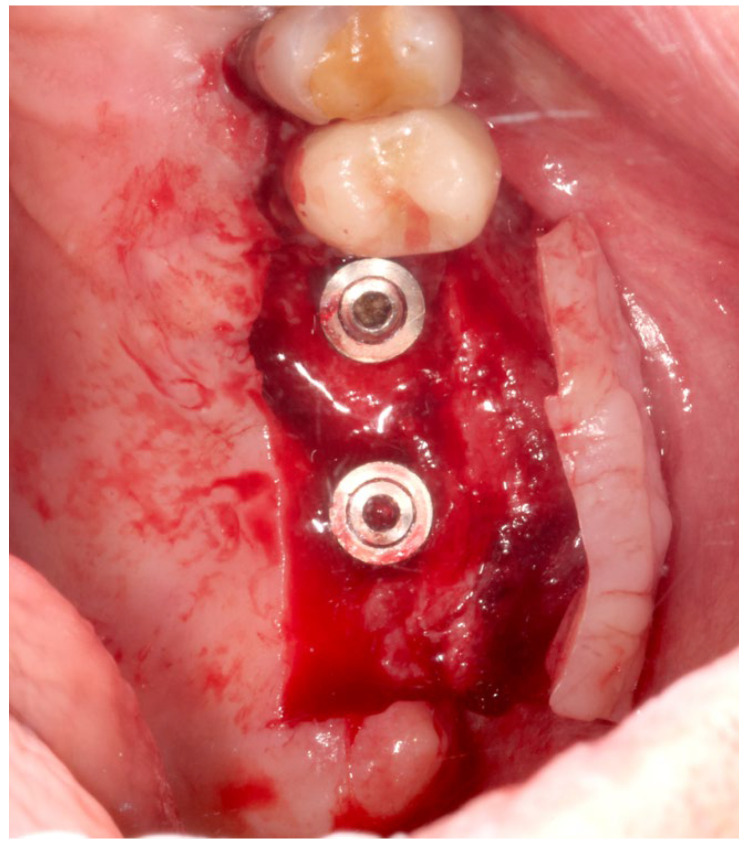
A partial-thickness flap was elevated to uncover the implants.

**Figure 3:**
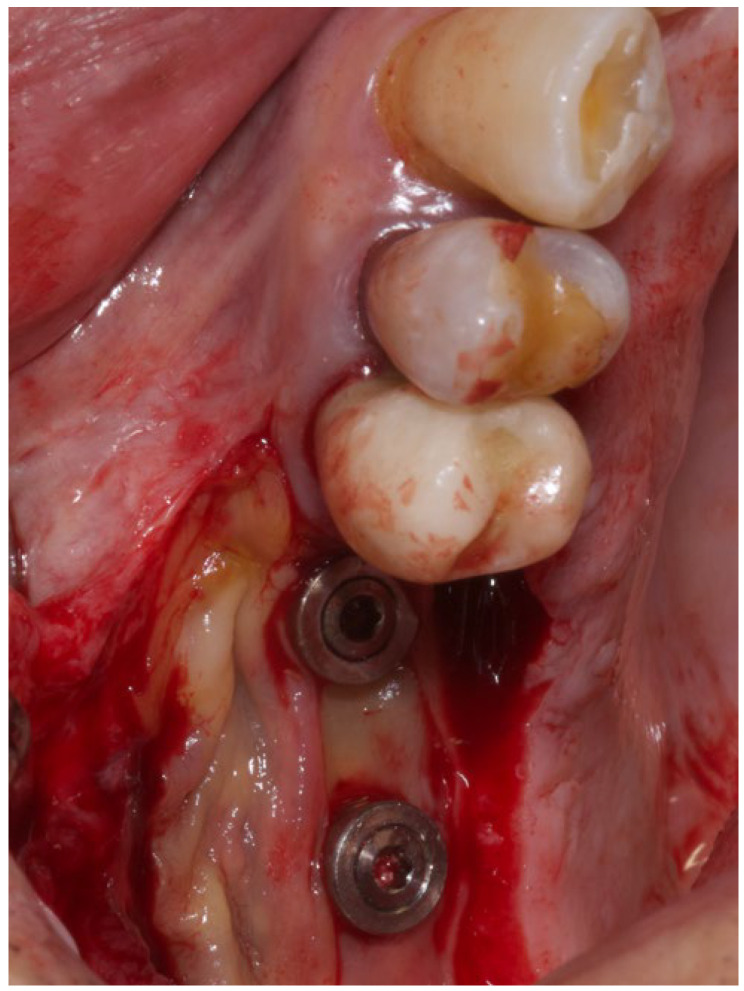
Positioning of L-PRF membranes on the palatal surgical wound.

The same experienced surgeon (MS) operated on all patients. The surgical procedures were performed during the implant uncovering stage, 20 weeks after implant placement. Before surgery, the patient rinsed with a 0.2% chlorhexidine solution (Dentosan 0.20, Johnson & Johnson, Pomezia, Italy) for 1 min. Following local anesthesia with mepivacaine 2% (Mepivacaina Pierrel Pharma srl, Capua, Italy), two vertical incisions were made, on the buccal side, mesially and distally to the mucosa of the edentulous area covering the fixtures, extending five millimeters beyond the muco-gingival junction of the adjacent teeth. The incisions were continued on the palatal side of the edentulous area in such a way as to extend for 4 mm into the keratinized mucosa; the ends of the parallel incisions were connected by a further horizontal incision on the palatal side. Subsequently a partial-thickness flap was elevated from the palatal edge to beyond the muco-gingival junction on the buccal side using a 15-C blade (Hu-Friedy, Milan, Italy) (Figure 2). The connective tissue above the cover screws was removed with a curette, and the healing abutment was positioned after filling the internal part of the fixture with 1% chlorhexidine gel (Corsodyl gel^®^, GlaxoSmithKline Consumer Healthcare S.p.A., Baranzate, Italy) [16]. A double layer of L-PRF membrane was then applied to the palatal surgical wound (Figure 3) and stabilized by compressive sutures. The flap was positioned buccally using a 3-0 non-absorbable silk suture (Ethicon Perma-Hand, Johnson & Johnson Medical Spa, Pomezia, Italy) fixed to the periosteum.

### 2.4. Post-Surgical Procedures

Patients were instructed to control post-operative pain with oral Ketoprofen 80 mg, to be used only if necessary. Plaque control was maintained using a 0.2% chlorhexidine digluconate solution (Dentosan 0.20, Johnson & Johnson, Pomezia, Italy) twice a day for 2 weeks after surgery. Fourteen days after surgery, sutures were removed, and the mechanical oral hygiene instructions were reinforced. All patients were recalled once a week for the first 4 weeks after surgery.

### 2.5. Wound Healing and Patient Morbidity

**Figure 4:**
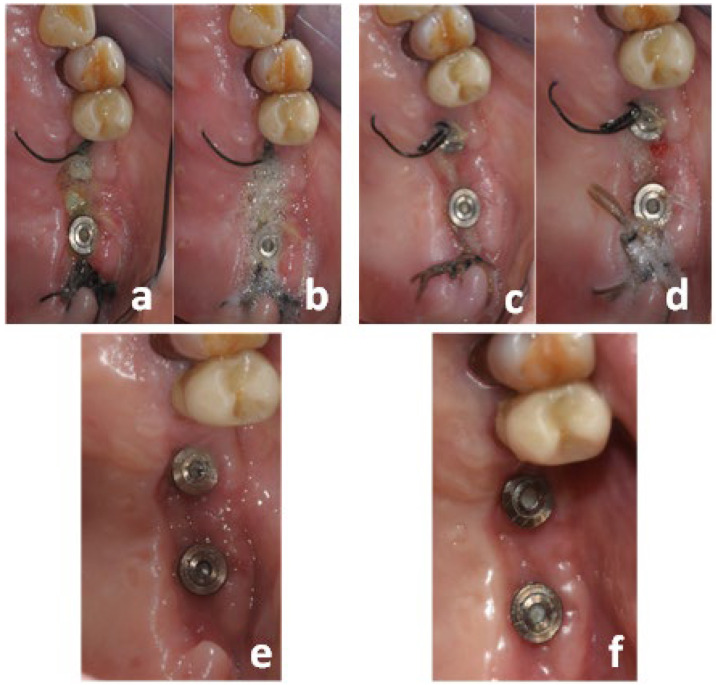
Peroxide test. (**a**,**b**): Week 1 examination. (**c**,**d**): Week 2 examination. (**e**): Week 3 examination: CWE obtained, no bubbles observed. (**f**): Week 4 examination.

The primary outcome of this study was the complete re-epithelialization of the surgical wound (CWE) after bandage with L-PRF membranes. Secondary outcomes were: (1) Post-operative discomfort (D); (2) Changes in the patient’s feeding habits (CFH); (3) The use of analgesics in the first week (AU); and (4) An alteration of sensitivity around the wound area (AS). Patients were instructed to use ketoprofen only in the case of relevant pain; the use of other pain medications was not permitted. The patients were also instructed to write down the number of doses of 80 mg of ketoprofen taken on a special form that had been given to them at the end of the surgical session. This study participants, who did not respect these requirements, were excluded from the experimental protocol. AU was calculated based on the sum of the analgesic doses administered to the patients at the end of the first week in order to indirectly quantify the patient’s pain. The CWE was clinically evaluated at the end of each week by a peroxide test; 3% H_2_O_2_ was applied to the healing area through a syringe; the possible appearance of bubbles would suggest incomplete re-epithelialization of the site, based on the principle that the catalase enzyme, present in exposed connective tissue, can react with H_2_O_2_ to produce water and oxygen (Figure 4). CWE was recorded as a dichotomous variable (Yes/No).

#### Statistical Analyses

The healing times were recorded as counts of re-epithelialized patients in each follow-up week. The parameters in VAS scale D, AS, and CFH were analyzed as pre/post-comparisons in the first two weeks by means of logistic ordinal mixed models, while the AU was estimated by means of a logistic ordinal model with the intercept predictor alone. A sensitivity analysis reanalyzing D, AS, and CFH using Wilcoxon’s Signed Rank test, paired Cliff’s delta test, and Linear Mixed Model Regression was performed, along with an estimation of AU by means of the Hodges-Lehmann estimator and the Arithmetic Mean. The R version 4.2.2 (31 October 2022 ucrt, R Core Team, Vienna, Austria) software package was used. D and CFH were assessed at each follow-up by representing the intensity of the event on a 100 mm visual analog scale (VAS) divided into 10 segments of 10 mm each and numbered from 0 to 10 (interview with VAS scale). In order to evaluate the pain intensity and change in eating habits resulting from the presence of the surgical wound, the patient had to indicate a value on the scale (0 to 10) [17]. AS was scored (0 to 10) by asking the patient to compare the sensitivity he/she had when the surrounding areas of the wound were touched by the tip of a periodontal probe and comparing this feeling to the feeling that he/she felt on the other side of the palate.

## 3. Results

No dropouts or post-operative complications occurred in this study, and the FMPS and FMBS remained <20% throughout the entire follow-up. Table 1 summarizes the results of this study. CWE occurred in 5 patients (25%) by the end of the second week, while the remaining 15 (75%) achieved CWE by the end of the third week. For D and CHF, a statistically significant improvement was recorded for all cases between the first and second weeks. However, for AS, no difference was reported between 7 and 14 days by the CLMM model test (we guess for insufficient power, being smaller the effect), while it was detected by the sensitivity analysis (paired Cliff’s test and linear mixed model). By the third week, all patients showed normal sensitivity, normal eating habits, and a cessation of discomfort. As regards the consumption of analgesics, only 2 patients recorded 5 administrations of ketoprofen during the first week; from the second week on, no analgesics had been taken.

From the third week on, all parameters returned to pre-surgical values.

## 4. Discussion

The aim of our study was to evaluate the efficacy of the L-PRF palatal bandage in favoring secondary intention healing of the surgical wound resulting from two-stage implant exposure while also evaluating patient-related outcomes of the surgical procedure. Our results demonstrated that within 2 weeks, 5 of 20 (25%) surgical sites presented CWE, while within 3 weeks, all sites achieved complete healing. Likewise, all secondary outcomes showed favorable evolution within 2 weeks, and all patients reported no D or CFH after the second week. Furthermore, after the first week, no patient reported the use of analgesics. As far as we are aware, there is no data in the literature about the time needed for CWE resulting from implant uncovering using APF. Data deriving from the clinical experience of our research group show that CWE after APF, using a bandage with an absorbable gelatin sponge, occurs in the majority of cases (70%) after 4 weeks (unpublished data). Using these data as a basis for comparison, the results obtained in this study show relevant clinical advantages deriving from bandage of the palatal wound using L-PRF. The healing times of the palatal wound healing that we have observed empirically after APF for implant uncovering, after bandage with absorbable gelatin sponge, are extremely similar to those of a palatal wound after free gingival graft harvesting treated in the same way. The clinical characteristics of the two palatal wounds are also very similar. Therefore, it would appear legitimate to use periodontal literature on free gingival graft harvesting in the discussion of our results. ARP, used as a fixture uncovering technique, is an easy-to-perform surgical procedure that predictably creates an adequate amount of keratinized tissue around maxillary implants and, at times, also around mandibular implants [7]. However, this surgical approach implies healing by secondary intention of an area palatal to the uncovered fixture, which could make the patient’s postoperative period uncomfortable, exposing them to the risk of prolonged bleeding. In our study, in order to overcome this drawback, the application of a double-layer L-PRF membrane enhanced the healing processes of the surgical wound around the implants. There are currently no studies in the literature that have tested the efficacy of L-PRF associated with APF in implant uncovering procedures. Femminella et al. [11], however, evaluated the usefulness of L-PRF in the management of the palatal wound from a free gingival graft (FGG) donor site by comparing it with that of a conventional hemostatic material. Using the same evaluation parameters, the Authors reported CWE at 2 weeks in 35% of patients in the L-PRF-treated group and complete healing of all sites within 3 weeks. These results are comparable to those obtained in the present study; conversely, for D and CFH parameters, the group treated with L-PRF in Femminella et al.’s study reported VAS scores higher than ours. This difference could be explained by the different typology and depth of the investigated wounds. In fact, the APF wounds in our study were more superficial than those obtained following a FGG harvest, being approximately 2 mm thick. Also in the study by Ozcan et al. [18], which evaluated the utility of a PRF dressing in the management of the palatal wound after FGG harvesting, the worse results reported for CFH compared to ours are probably attributable to the greater depth of the wound. In fact, Ozcan et al. found a return to normal feeding habits between the third and fourth weeks. Patarapongsanti et al. compared patient morbidity and healing outcomes after FGG harvesting from the palate using platelet-rich fibrin (PRF) or oxidized regenerated cellulose (ORC) [19]. Although the Authors reported, in the L-PRF group, a CWE in week 3, as in the present study, the percentage of patients who achieved CWE by week 2 was higher (88.8%) than the percentage we observed (25%). This result could be explained by the low thickness of the harvested graft (<2 mm) in Patarapongsanti’s study and by the smaller size of the wound area compared to that in the present study. In the ORC-treated group, CWE was achieved during the fourth week, showing less favorable results than those of our study. An accelerated healing of the palatal wound after FGG harvesting is also confirmed by other Authors, who support the results obtained in the present study [20,21,22]. In contrast, Belkhede et al. [23], making a comparative evaluation of PRF bandage versus gelatin sponge in the healing of palatal wounds from FGG donor sites, found faster CWE in the gelatin sponge-treated group, reporting a CWE of the L-PRF-treated group only at the third week. This difference, however, could be attributed to the small sample size recruited in the study, the lack of standardization in the size of the palatal wounds, and the different types of wounds considered. Although the present study presents a low number of clinical cases, the similarity of our results with those obtained from most of the randomized controlled studies evaluating L-PRF in the bandage of palatal wounds from FGG harvest allows us to hypothesize that the bandage with L-PRF is superior to that made with traditional materials (i.e., gelatin sponge or oxidized regenerated cellulose), even in the case of palatal wounds deriving from the use of APF for endosseous fixture uncovering.

Moreover, our results are consistent with the biological premises deriving from the nature and characteristics of L-PRF [11,19]. First, the angiogenic stimulus induced by the fibrin matrix of L-PRF is mediated by a series of soluble factors contained therein, such as vascular endothelial growth factor, platelet-derived growth factor, and fibroblast growth factor [12,24]. In addition, the fibrin membrane is able to release several chemokines, such as RANTES, Interleukin-8, Macrophage Inflammatory Protein-1, Epithelial Neutrophil-Activating Protein 78, and Monocyte Chemotactic Protein-3, which attract leukocytes, activate other platelets, and also modulate the production of inflammatory molecules by endothelial cells [25]. In the wound healing phase, the epithelial cells lose their basal and apical polarity and produce basal and lateral extensions among the sides of the wound [26]. L-PRF also acts as a scaffold for the healing of injured tissue by providing a transient matrix consisting of fibrinogen, fibronectin, tenascin, and vitronectin [26,27]. Furthermore, fibrin, fibronectin, PDGF, and TGF-β are essential for the modulation of integrin expression, for fibroblastic proliferation, and for their migration within the wound [25]. The low number of painkillers taken in the first week could only be explained by the properties of the L-PRF multilayer membrane acting as an instrument of isolation from the surrounding environment and protection of the sensory structures exposed by the surgical procedure [11]. Among the factors influencing palate healing, we undoubtedly consider the patient’s systemic conditions, post-operative dietary habits, periodontal status, and the pain scale before manipulation to identify those with heightened or reduced pain thresholds. Specifically, patients with coagulation disorders (a history of hemophilia, von Willebrand disease), those currently undergoing anticoagulant therapy, and individuals with altered healing patterns (such as type 1 diabetes mellitus) were excluded to mitigate the risk of post-operative bleeding. This precaution includes avoiding additional hemostatic drugs. Experiencing profuse bleeding during oral surgery presents a considerable challenge, potentially impairing the operator’s visibility and leading to prolonged post-operative bleeding or hematoma formation. Such conditions heighten the risk of bacterial infections [28]. Eating habits are a significantly debated factor in periodontal surgery. Previous studies indicate that a diverse range of dietary components, including macronutrients and micronutrients, play a crucial role in maintaining optimal periodontal health and have the potential to expedite oral wound healing following periodontal procedures [29]. Our findings underscore the importance of providing patients with detailed oral and written postoperative instructions. These instructions should encompass adherence to a soft food diet and avoidance of excessive physical exertion, brushing, flossing, or any other activities that could cause trauma near the surgical sites. Patients should also refrain from vigorous mouth rinsing, smoking, negative pressure (such as suction or expectoration), and, if approved by a physician, discontinuation of anticoagulant therapy during the 2–3 weeks following surgery [30]. Moreover, the patients enrolled were all periodontally healthy, so as not to have problems relating to the active phase of the disease, or in any case, patients who did not present muco-gingival defects that could affect the healing of the palate after APF. Finally, patients were reminded to specifically report pain experienced in the palate during the Visual Analog Scale (VAS) recording, excluding any pain felt before the operation. This instruction is given considering that the enrolled patients are individuals who do not regularly use analgesic drugs and do not have issues related to pain [31].

The main limitation of the present study is the lack of a control group, which does not permit us, with a scientific method, to prove the greater efficacy of the treatment here proposed in comparison to conventional bandages. Therefore, our promising preliminary results need validation by randomized controlled trials. Currently, our Research Group is carrying out a clinical trial based on that experimental protocol.

## 5. Conclusions

The APF for two-stage maxillary dental implant uncovering is a technique that predictably allows increasing, or creating, keratinized mucosa. However, this procedure produces a palatal wound that heals by secondary intention, with a definite increase in postoperative morbidity. The results that we obtained seem to confirm the biological premises underlying the present study. The bandage of the palatal wound by L-PRF accelerates the complete epithelialization of the wound compared to the healing times usually and empirically observed with the use of traditional bandages (i.e., fibrin sponge). At the same time, post-operative morbidity appears to be low. Furthermore, our results show good agreement with those of the literature on the healing of palatal wounds after FGG harvest medicated with L-PRF or traditional bandages.

## Figures and Tables

**Table 1:** Re-epithelialization times, analgesic consumption amounts, and patient-related VAS scale parameters Mean.class is the average of the estimates of the probability distribution of each rating in the ordinal models, i.e., the averages of the probabilities of integers, as provided by the mode = “mean.class” parameter of the R function emmeans::emmeans estimating the output of the clmm and clm functions of the ordinal package.

Week	Re-Epithelialized Patients	Discomfort	Sensitivity Alteration	Feeding Habits Change	Analgesic Usage
		Mean.Class ± SE (95% CI)	Mean.Class ± SE (95% CI)	Mean.Class ± SE (95% CI)	Mean.Class ± SE (95% CI)
1	-	3.01 ± 0.54 (2.90 to 3.12)	1.42 ± 0.268 (0.895 to 1.95)	3.09 ± 0.679 (1.755 to 4.42)	1.1 ± 0.0671 (0.96 to 1.23)
2	5	1.00 ± 0.00 (1.0 to 1.0)	1.05 ± 0.799 (0.898 to 1.21)	1.09 ± 0.184 (0.726 to 1.45)	

## Data Availability

All data generated or analyzed during this study are included in this published article.
